# Supplementary material for: The diagnostic value of core needle biopsy in cervical cancer: A retrospective analysis
Source: PLoS One. 2022 Jan 5;17(1):e0262257. doi: 10.1371/journal.pone.0262257 (PMC8730459; doi:10.1371/journal.pone.0262257)
Supplement: Attachment — Submitted filename: Response to Reviewers.docx [file pone.0262257.s002.docx]

Massimilinao Lia

Department of Gynecology and Obstetrics

University Hospital Leipzig

Liebigstr. 20a

D-04103 Leipzig

E-Mail: massimiliano.lia@medizin.uni-leipzig.de

25^nd^ August 2021

Rebuttal letter

Dear editor, dear reviewer,

thank you very much for your supportive feedback which consistently improves this article.

We have integrated the changes proposed by you in the manuscript and, as requested, an answer to every point raised by you has been added to this rebuttal letter (answers are written in bold) and some are also addressed in the revised cover letter.

1. Please ensure that your manuscript meets PLOS ONE's style requirements, including those for file naming. The PLOS ONE style templates can be found at

https://journals.plos.org/plosone/s/file?id=wjVg/PLOSOne_formatting_sample_main_body.pdf and

https://journals.plos.org/plosone/s/file?id=ba62/PLOSOne_formatting_sample_title_authors_affiliations.pdf

**Done**

2. Please state whether patients provided written informed consent in the ethics statement in the manuscript Methods."

**Line 93**

3. In your Methods section, please provide additional information about the participant recruitment method and the demographic details of your participants. Please ensure you have provided sufficient details to replicate the analyses such as the recruitment date range (month and year).

**Recruitment: Line 94-98**

**Details partecipants: Line 159-166 and Table 1**

4. We note that you have indicated that data from this study are available upon request. PLOS only allows data to be available upon request if there are legal or ethical restrictions on sharing data publicly. For information on unacceptable data access restrictions, please see <http://journals.plos.org/plosone/s/data-availability#loc-unacceptable-data-access-restrictions>.

**The sharing of raw data, even anonymized, is not supported by the ethical committee of the university hospital in Leipzig (Stephanstraße 9A.1, 04103 Leipzig, Germany), as it contains sensitive patient information including medical history and oncological details. Thus, sharing of raw data was not part of the written consent form signed by the patients.**

In your revised cover letter, please address the following prompts:

a) If there are ethical or legal restrictions on sharing a de-identified data set, please explain them in detail (e.g., data contain potentially identifying or sensitive patient information) and who has imposed them (e.g., an ethics committee). Please also provide contact information for a data access committee, ethics committee, or other institutional body to which data requests may be sent.

**Done**

b) If there are no restrictions, please upload the minimal anonymized data set necessary to replicate your study findings as either Supporting Information files or to a stable, public repository and provide us with the relevant URLs, DOIs, or accession numbers. Please see http://www.bmj.com/content/340/bmj.c181.long for guidelines on how to de-identify and prepare clinical data for publication. For a list of acceptable repositories, please see http://journals.plos.org/plosone/s/data-availability#loc-recommended-repositories.

**Minimal anonymized data has been uploaded**

We will update your Data Availability statement on your behalf to reflect the information you provide.

5. We noted in your submission details that a portion of your manuscript may have been presented or published elsewhere.

"The abstract of this manuscript has been submitted to the 2021 ASCO Annual Meeting. The abstract submitted to the 2021 ASCO Annual Meeting is slightly different, due to different formatting guidelines, compared with the abstract of the manuscript herby submitted to PLOS ONE. However, title, authors and results are exactly the same, making it clear for every reader that the two abstracts describe the same study. Furthermore, the ASCO guidelines allow the submission of a pre-published abstract. Finally, we feel the whole study should be accessible, since the abstract alone can´t possibly reflect all aspects of the results of this study. "

Please clarify whether this conference proceeding was peer-reviewed and formally published. If this work was previously peer-reviewed and published, in the cover letter please provide the reason that this work does not constitute dual publication and should be included in the current manuscript.

**It has been included in the revised cover letter.**

6. Thank you for stating the following in the Competing Interests section:

"I have read the journal's policy and the authors of this manuscript have the following

competing interests: MH and BW received payments from the non-profit-organization

“Stiftung gynäkologische Onkologie”.

BA received honoraria from Pfizer Inc, Roche AG, Novartis AG, AstraZeneca PLC,

Amgen Inc and Daiichi Sankyo Co Ltd.

LCH, PS and ML have no conflicts to disclose."

Please confirm that this does not alter your adherence to all PLOS ONE policies on sharing data and materials, by including the following statement: "This does not alter our adherence to PLOS ONE policies on sharing data and materials.” (as detailed online in our guide for authors http://journals.plos.org/plosone/s/competing-interests). If there are restrictions on sharing of data and/or materials, please state these. Please note that we cannot proceed with consideration of your article until this information has been declared.

Please include your updated Competing Interests statement in your cover letter; we will change the online submission form on your behalf.

**It has been included in the revised cover letter.**

Please know it is PLOS ONE policy for corresponding authors to declare, on behalf of all authors, all potential competing interests for the purposes of transparency. PLOS defines a competing interest as anything that interferes with, or could reasonably be perceived as interfering with, the full and objective presentation, peer review, editorial decision-making, or publication of research or non-research articles submitted to one of the journals. Competing interests can be financial or non-financial, professional, or personal. Competing interests can arise in relationship to an organization or another person. Please follow this link to our website for more details on competing interests: http://journals.plos.org/plosone/s/competing-interests

**Reviewers' comments:**

Abstract/Introduction

- LVSI and grade have a prognostic impact, but can the Authors clarify how this can impact surgery and therapeutic decision?

**The role of LVSI in therapeutic decision making has been clarified in line 261-267. The role of grade is less clear, but evidence has been discussed in line 280-284.**

- Can the Authors clarify what is the primary endpoint of the study? How was the “performance” assessed?

**The performance of histological subtype was performed by sensitivity and specificity**

**For lymphovascular space invasion and tumor-grading we calculated the sensitivity, specificity, accuracy, positive/negative predictive value and the kappa-value (chance corrected agreement between CNB and hysterectomy-specimen). This has been clarified in the methods section (line 136-144).**

- Authors at Line 70 state that “no studies evaluating the accuracy of CNB in cervical cancer have been published”; however, an initial description of such technique has been reported in the following study: Mascilini F, Quagliozzi L, Moro F, et al. Role of transvaginal ultrasound-guided biopsy in gynecology. Int J Gynecol Cancer. 2020;30(1):128-132. doi:10.1136/ijgc-2019-000734

**You are absolutely right and we have included this article as references in the introduction (line 69-72).**

Methods

- Authors should clearly state whether the study was prospective or retrospective as it is not clear in the methodology

**This has been included in the methodology (line 77).**

- Can the Authors specify it in the methods the criteria to take more than one core needle biopsy for each patient?

**The number of biopsies were determined in order to ensure a proper tissue sample for a complete histological classification of the tumor. Usually, the number of biopsies performed was higher than one.**

- Line 82: was classification from FIGO 2018 or 2009 used? Can the Authors clarify in the text?

**The classification of 2009. We included this in the text (line 102).**

Discussion

- Line 214: Authors state that CNB can be useful to determine peri neural infiltration, but they did not report it in the results and they did not report reference for this statement

**This is a general statement about the CNB not referring exclusively to cervical cancer. Perineural infiltration was not analysed in this study, as it is not routinely assessed in CNB-tissue in cervical cancer. The reviewer is completely right to criticize the lack of a proper citation or study data backing this statement. Thus, we deleted this part as it is superfluous for this study.**

- Discussion could be shortened to 2/2.5 pages.

**Done.**

While revising your submission, please upload your figure files to the Preflight Analysis and Conversion Engine (PACE) digital diagnostic tool, https://pacev2.apexcovantage.com/. PACE helps ensure that figures meet PLOS requirements. To use PACE, you must first register as a user. Registration is free. Then, login and navigate to the UPLOAD tab, where you will find detailed instructions on how to use the tool. If you encounter any issues or have any questions when using PACE, please email PLOS at figures@plos.org. Please note that Supporting Information files do not need this step.

Massimiliano Lia
